# Stakeholder development of the Malaria Decision Analysis Support Tool (MDAST)

**DOI:** 10.1186/1475-2875-11-S1-P15

**Published:** 2012-10-15

**Authors:** Zachary Brown, Randall Kramer, Clifford Mutero, Dohyeong Kim, Marie Lynn Miranda, Birkinesh Ameneshewa, Adriane Lesser, Christopher J Paul

**Affiliations:** 1 Organisation for Economic Co-operation and Development, Paris, 75775, France; 2 Nicholas School of the Environment, Duke University, Durham, North Carolina, 27708, USA; 3 Duke Global Health Institute, Duke University, Durham, North Carolina, 27708, USA; 4 School of Health Systems and Public Health, University of Pretoria, Pretoria, 0028, South Africa; 5 icipe, Nairobi, Kenya; 6 Department of Public Administration, North Carolina Central University, Durham, North Carolina, 27707, USA; 7 School of Natural Resources and Environment, University of Michigan, Ann Arbor, Michigan, 48109, USA; 8 WHO Regional Office for Africa, Harare, Zimbabwe

## Background

Although exceptional progress has been made towards controlling and eventually eliminating malaria from sub-Saharan Africa, recent efforts have sometimes faltered. Reasons for this include the development of resistance in parasites and vectors to current control strategies, volatile funding streams, and funding allocations which sometimes do not efficiently achieve the goals of project managers, policy makers, or citizens. The project described here implements an approach to evidence-based policy for malaria control using a decision analysis framework proposed by Kramer et al. [1]. The project consists of the stakeholder-driven implementation of that framework through the development of a Malaria Decision Analysis Support Tool (MDAST) in Kenya, Tanzania, and Uganda. Results from the project to date point towards large anticipated value from stakeholder-driven implementation of a tool such as MDAST at the policy, programmatic, and technical levels.

## Materials and methods

MDAST is an evidence-based framework to assess health, social, economic, and environmental outcomes that can result from alternative malaria control strategies. It was developed through an iterative process consisting of the following activities:

• Recruit stakeholders who are experts and decision makers in malaria control policy across governmental and academic sectors in Kenya, Tanzania, and Uganda.

• Elicit influence diagrams from stakeholder workshops about factors determining outcomes of different interventions.

• Review field and modeling research on the short- and long-run effectiveness of dominant malaria control interventions.

• Develop a rapidly deployable, open-access, and customizable software tool using the Analytica^®^ Decision Analysis Platform. The software combines stakeholders’ influence diagrams and our review of scientific research.

• Demonstrate and elicit stakeholder feedback on the tool through hands-on workshops.

• Refine the software to reflect stakeholder feedback on scientific content and ease-of-use/interpretability.

## Results

MDAST permits the analysis of risks that (a) were identified by stakeholders as important in determining effectiveness of different policies, and (b) have not previously been combined in a practical, flexible tool. These features include the dynamic selection of insecticide resistance in the mosquito population, as well as options of different long lasting insecticidal net (LLIN) distribution mechanisms (mass distribution or voucher-subsidized).

Through anonymous written surveys during the workshops, participants indicated high levels of enthusiasm for using the tool, and provided essential feedback on how it can be improved (e.g., additional IRS insecticides and the capacity to rotate them, and better representation of larviciding), and identified barriers to implementation (e.g., context-specific data for calibrating MDAST to reflect local conditions).

## Conclusions

The MDAST project demonstrates the need for compact systems to exchange evidence between scientific, policy, and program management communities for analyzing the potential outcomes of alternative policy decisions. MDAST works to address this need in participating countries. Continued engagement with stakeholders, and with scientists producing the primary research on which this tool relies, is necessary to complete implementation of MDAST and develop extensions of similar tools to additional locations and situations.
